# Efficacy of common disinfection processes against infective spores (arthroconidia) and mycelia of *Microsporum gallinae* causing avian dermatophytosis

**DOI:** 10.14202/vetworld.2022.1413-1422

**Published:** 2022-06-08

**Authors:** Eakachai Thongkham, Sucheeva Junnu, Glenn Neville Borlace, Suwit Uopasai, Jareerat Aiemsaard

**Affiliations:** 1 Division of Pharmacology and Toxicology, Faculty of Veterinary Medicine, Khon Kaen University, Khon Kaen, 40002 Thailand; 2 Division of Livestock Medicine, Faculty of Veterinary Medicine, Khon Kaen University, Khon Kaen, 40002 Thailand; 3 Department of Pharmaceutical Chemistry, Faculty of Pharmaceutical Sciences, Khon Kaen University, Khon Kaen, 40002 Thailand; 4 Division of Anatomy, Faculty of Veterinary Medicine, Khon Kaen University, Khon Kaen, 40002 Thailand

**Keywords:** arthroconidia, avian dermatophytosis, disinfection processes, *Microsporum gallinae*

## Abstract

**Background and Aim:**

*Microsporum gallinae* is the major dermatophyte species that causes avian dermatophytosis. Disinfection plays an important role in controlling and preventing dermatophytosis; however, information about the effect of common disinfection processes on *M. gallinae* is limited. This study aimed to investigate the disinfection efficacy of ultraviolet (UV) irradiation, heat treatment, detergents, and germicides against infective spores (arthroconidia) and vegetative mycelia of *M. gallinae*.

**Materials and Methods:**

The minimum inhibitory and minimum fungicidal concentrations of benzalkonium chloride, chlorhexidine, ethanol, formaldehyde, glutaraldehyde, hydrogen peroxide, phenol, povidone-iodine, and sodium hypochlorite germicides against arthroconidia and mycelia of *M. gallinae* American type culture collection (ATCC) 90749 were determined by broth microdilution. Time-kill assays were used to determine the fungicidal efficacy of moist heat treatment, UV irradiation, commercially available detergents, and germicides.

**Results:**

There were no significant differences between the arthroconidia and mycelia growth stages of *M. gallinae* ATCC 90749 in the magnitude of the log_10_ cell reductions in the number of viable fungal cells induced by the disinfection treatments (all p > 0.05). Moist heat treatment at 40°C did not reduce the number of viable fungal cells at any time (1–60 min); however, treatment at 50°C for 25 min and either 60°C or 80°C for 5 min eliminated > 99.999% of viable fungal cells. Irradiation of fungal cultures with UVC and UVB at doses higher than or equal to 0.4 and 0.8 J/cm^2^, respectively, resulted in a 5-log_10_ reduction in the number of viable fungal cells, whereas UVA only reduced the number of viable fungal cells by < 2-log_10_ up to a dose of 1.6 J/cm^2^. All the tested detergents demonstrated minimal fungicidal effects with < 1-log_10_ reductions in the number of viable fungal cells at concentrations up to 8% w/v. All of the tested germicides eradicated the fungus after treatment for 1 min at 1–1000× minimum inhibitory concentration (MIC), except for hydrogen peroxide, which was not fungicidal after treatment for 20 min at 100× MIC.

**Conclusion:**

Moist heat treatment at temperatures greater than or equal to 50°C, UVC and UVB irradiation at doses higher than or equal to 0.4 and 0.8 J/cm^2^, respectively, and treatment with all tested germicides except hydrogen peroxide can be considered effective processes for disinfecting the fungus *M. gallinae* from the equipment employed in poultry farming. In contrast, commercially available detergents are not suitable for use as *M. gallinae* disinfectants.

## Introduction

Avian dermatophytosis or favus is a sporadic superficial mycosis observed in poultry that is mainly caused by the fungal dermatophyte *Microsporum gallinae* [1]. *M. gallinae* is common in gallinaceous birds and other poultry such as ducks and pigeons and has also been shown to cause disease in mammals, including dogs, cats, mice, squirrels, cattle, and monkeys [2, 3]. It is a member of the zoophilic fungi, which are a public health concern because they can cause severe acute skin inflammation in humans exposed to infected animals [4, 5].

In avian dermatophytosis, infective fungal spores of *M. gallinae*, termed arthroconidia, adhere to normal poultry skin and dermatitis develops on the epidermis, and hair follicle and shaft. The characteristic white scaly lesions are usually found on featherless areas, such as the comb, wattle, and shanks, but may spread to other parts of the body, making the animal prone to itching. In severe cases, feather follicles may be destroyed and animals may show systemic signs of illness, such as depression and loss of appetite. The disease is usually chronic and progressive but not fatal. Nevertheless, avian dermatophytosis causes stress to the animal, decreasing its quality of life as well as the quality of the carcass, possibly affecting the economic productivity of broilers, laying hens, and breeder chickens [6, 7]. *M. gallinae* is transmitted through direct contact with infected animals or contaminated fomites, such as poultry litter, clothes, equipment, and tools [7]. Therefore, the disinfection of poultry farming-related equipment and use of effective detergents and germicides for cleaning the environment and equipment as well as for maintaining personal hygiene are crucial to reduce the transmission of *M. gallinae* among chickens in poultry farms, from poultry to other animals, and from infected animals to humans [8].

Heat, ultraviolet (UV) irradiation, detergents, and disinfectants are commonly used to clean and disinfect equipment, clothing, and people and can eradicate fungi. However, the effectiveness of disinfection processes can vary depending on the type of fungi [9, 10].

To the best of our knowledge, no previous study has tested the efficacy of disinfection processes in decontaminating the fungus *M. gallinae*. Therefore, traditional cleaning and disinfection practices used in poultry husbandry may be suboptimal. This study aimed to investigate the disinfection efficacy of UV irradiation, heat treatment, detergents, and germicides against infective spores (arthroconidia) and vegetative mycelia of *M. gallinae*.

## Materials and Methods

### Ethical approval

This study used *in vitro* experiments, so ethical approval was not necessary.

### Study period and location

This study was conducted from May 2021 to January 2022 at the Faculty of Veterinary Medicine, Khon Kaen University, Thailand.

### Materials

*M. gallinae* strain American type culture collection (ATCC) 90749 was obtained from the ATCC-Corporate Office, University Boulevard Manassas, Virginia. Sabouraud dextrose agar (SDA) and Sabouraud dextrose broth (SDB) were supplied by Becton Dickinson, Grenoble, France. Roswell Park Memorial Institute (RPMI)-1640 medium was from Sigma-Aldrich, Germany. Germicides were supplied by Sigma-Aldrich, Germany (benzalkonium chloride and chlorhexidine); RCI Labscan Ltd., Thailand (ethyl alcohol); Loba Chemie Pvt. Ltd., India (formaldehyde, glutaraldehyde, and phenol); Qchemical Co., Ltd., Thailand (hydrogen peroxide); Leopard Medical Brand Co., Ltd., Thailand (povidone-iodine); and Chemipan Corporation Co., Ltd., Thailand (sodium hypochlorite). The commercially available detergents: Downy^®^ powdered laundry detergent (Procter and Gamble Trading [Thailand] Co. Ltd., Bangkok, Thailand); Seventh Generation™ liquid laundry detergent (Unilever Thai Trading Co. Ltd., Bangkok, Thailand); Protex™ liquid body soap (Colgate-Palmolive [Thailand] Co. Ltd., Chonburi, Thailand); Dettol^®^ liquid hand soap (Reckitt Benckiser [Thailand] Co. Ltd., Bangkok, Thailand); and Sunlight^®^ dishwashing liquid (Unilever Thai Trading Co. Ltd., Bangkok, Thailand) were purchased from local retailers. Lecithin was purchased from Mega Lifesciences Pty Ltd., Thailand; sodium thiosulfate from Elango Enterprises Pty Ltd., Australia; and polysorbate 80 from Ajax Finechem Pty Ltd., Australia.

### Fungal culture and preparation

Fungal arthroconidia and mycelia were prepared *in vitro* to control their quantity and quality. Arthroconidia were prepared by culturing *M. gallinae* ATCC 90749 on SDA (pH 5.6) plates at 37°C under 5% CO_2_ with 80% relative humidity in a water-jacketed CO_2_ incubator (Esco CelCulture^®^, Esco Micro Pte. Ltd., Singapore). After 14 days, 5 mL of phosphate-buffered saline (PBS) (pH 7.2) was added to the inoculated plates and fungal fragments were collected using a triangle-shaped glass rod spreader. The fungal suspension was double filtered through 10 layers of folded sterile gauze to separate the hyphae and arthroconidia. The presence of arthroconidia in the filtrate was confirmed under a light microscope (Olympus Optical Co., Ltd., Japan) at 400–1000× magnification. Fungal fragments that were < 4 mm in length with conspicuous detachment scars at both ends were considered arthroconidia. Mycelia were prepared by culturing *M. gallinae* ATCC 90749 in SDB in an Erlenmeyer flask at 30°C with continuous stirring for 5 days. The mycelial suspension was homogenized using a tissue grinder. The concentration of fungal suspensions used in the susceptibility tests was confirmed by aerobic plate counts [11, 12].

### Fungicidal efficacy of moist heat treatment

Arthroconidial and mycelial suspensions of *M. gallinae* ATCC 90749 were diluted with PBS to a final concentration of 1 × 10^5^ colony forming units (CFU)/mL and 1 mL aliquots were added to 1.5 mL microcentrifuge tubes. The fungal suspensions were incubated at 40, 50, 60, and 80°C in a temperature-controlled water bath (Gesellschaft für Labortechnik mbH, Germany). After 5, 10, 15, 20, 25, 30, 35, 40, 45, 50, 55, and 60 min, 1 mL of samples were inoculated onto SDA plates and incubated at 30°C for 96 h, before recording the number of fungal colonies. Fungal suspensions incubated at 30°C were used as the control [13]. Each experiment was performed in triplicate.

### Fungicidal efficacy of UV irradiation

Ten microliter samples of 1 × 10^7^ CFU/mL arthroconidial and mycelial suspensions of *M. gallinae* ATCC 90749 were dispensed onto 15 × 100 mm (height × diameter) sterile glass Petri dishes and allowed to air dry for 30 min in a 37°C incubator. The plates were then exposed to a UV lamp (Cole-Parmer Instrument Company Ltd., UK), generating UVA (365 nm), UVB (302 nm), and UVC (254 nm) radiation at doses of 0.1, 0.2, 0.4, 0.8, and 1.6 J/cm^2^. Following treatment, the fungal materials were resuspended with 1 mL of PBS, inoculated onto SDA plates, and incubated at 30°C for 96 h, before recording the number of fungal colonies. Fungal samples without UV exposure were used as the control [14]. Each experiment was performed in triplicate.

### Fungicidal efficacy of commercially available detergents

Time-kill assays were performed to evaluate the antifungal activity of commercially available detergents against *M. gallinae* ATCC 90749. Commercially available powdered laundry detergent, liquid laundry detergent, liquid body soap, liquid hand soap, and dishwashing liquid that are likely to be routinely used for cleaning poultry equipment and tools and for personal hygiene were selected, the active surfactant ingredients of which are given in Table-1. Briefly, 100 µL of *M. gallinae* ATCC 90749 arthroconidial and mycelial suspensions (1 × 10^7^ CFU/mL) were homogenously mixed with 900 µL of the detergent products to obtain final concentrations of 1%, 2%, 4%, and 8% w/v. After incubation at 30°C for 1, 2, 3, 4, 5, 10, 15, and 20 min, the mixtures were diluted with PBS to stop the reaction and obtain final dilutions of 10^−1^, 10^−2^, 10^−3^, and 10^−4^. Next, 1 mL of each dilution was inoculated onto SDA plates and incubated at 30°C for 96 h. The number of fungal colonies was recorded. Fungal suspensions without detergent were used as the control [11, 15]. Each experiment was performed in triplicate.

**Table 1:** The information on selected commercially available detergents.

Product	Trademark	Active surfactant
Powdered laundry detergent	Downy^®^	Sodium linear alkylbenzene sulfonate
Liquid laundry detergent	Seventh Generation^™^	Ethoxylated alcohol, sodium lauryl sulfate, sodium oleate
Liquid body soap	Protex^™^	Sodium laureth sulfate, potassium laurate, potassium myristate, cocamidopropyl betaine, potassium palmitate, potassium oleate
Liquid hand soap	Dettol^®^	Ammonium lauryl sulfate, sodium laureth sulfate, cocamide monoethanolamine
Dishwashing liquid	Sunlight^®^	Sodium linear alkylbenzene sulfonate, sodium laureth sulfate

### Antifungal efficacy of germicides

#### Broth microdilution method

Nine commonly used antiseptics and disinfectants, including benzalkonium chloride, chlorhexidine, ethanol, formaldehyde, glutaraldehyde, hydrogen peroxide, phenol, povidone-iodine, and sodium hypochlorite, were tested for antifungal activity against *M. gallinae* ATCC 90749 using the broth microdilution method. The assay was performed according to Clinical and Laboratory Standard Institute [16] guidelines with some modifications. Briefly, stock solutions of each germicide were prepared by dilution with an appropriate solvent and then diluted to working solutions with sterile deionized water (Table-2). Next, 50 µL of RPMI-1640 broth was added to all wells of a 96-well round-bottomed microtiter plate. The working solution of each germicide was added to each well of the first column and serial 2-fold dilutions were performed from the 1^st^ to the 10^th^ column. Subsequently, 50 µL of *M. gallinae* ATCC 90749 arthroconidial or mycelial suspension (1 × 10^4^ CFU/mL) was added into wells from the 1^st^ to 11^th^ columns. The wells of the 11^th^ and 12^th^ columns were used as positive and negative growth controls (broth with fungal suspension and broth only, respectively). The plates were incubated at 30°C for 96 h. The minimum inhibitory concentration (MIC) was determined from the lowest concentration of germicide inhibiting visible growth after 96 h of incubation. Subsequently, 10 µL samples from the wells with no visible growth were inoculated onto SDA plates and incubated at 30°C for 96 h. The minimum fungicidal concentration (MFC) was determined from the lowest concentration of germicide that inhibited growth on the SDA plates. Each experiment was performed in triplicate.

**Table 2:** Germicide concentration ranges and solvents.

Agent	Solvent	Stock solution concentration	Working solution concentration	Tested concentration
Benzalkonium chloride	Deionized water	10 mg/mL	100 μg/mL	0.0488–25 μg/mL
Chlorhexidine	Dimethyl sulfoxide	10 mg/mL	100 μg/mL	0.0488–25 μg/mL
Ethyl alcohol	Not dilute	1,000 μL/mL	1,000 μL/mL	1.6–800 μL/mL
Formaldehyde	Deionized water	10 mg/mL	1,600 μg/mL	0.0781–400 μg/mL
Glutaraldehyde	Deionized water	10 mg/mL	1,600 μg/mL	0.0781–400 μg/mL
Hydrogen peroxide	Deionized water	120 mg/mL	102,400 μg/mL	5–25,600 μg/mL
Phenol	Ethyl alcohol	500 mg/mL	204,800 μg/mL	100–51,200 μg/mL
Povidone-iodine	Deionized water	100 mg/mL	51,200 μg/mL	25–12,800 μg/mL
Sodium hypochlorite	Deionized water	100 mg/mL	51,200 μg/mL	25–12,800 μg/mL

#### Time-kill assay

To perform the time-kill assay, 100 µL of *M. gallinae* ATCC 90749 arthroconidial suspension (1 × 10^7^ CFU/mL) was homogeneously mixed with 900 µL of each germicide to give final concentrations of 1-1000 times their respective MICs. After incubation at 30°C for 1, 2, 3, 4, 5, 10, 15, and 20 min, the mixture was diluted with a neutralizing solution (0.6% w/v sodium thiosulfate, 0.5% w/v polysorbate 80, and 0.07% w/v lecithin in PBS) to final dilutions of 10^−1^, 10^−2^, 10^−3^, and 10^−4^. Subsequently, 1 mL sample of each dilution was inoculated onto SDA plates and incubated at 30°C for 96 h. The number of fungal colonies was recorded. Fungal suspension with the neutralizing solution was used as the control [11, 15]. Each experiment was performed in triplicate.

### Statistical analysis

The normality of the data was assessed through the Shapiro–Wilk test. After each disinfection treatment, differences in the reduction of viable arthroconidia and mycelia were compared using the Mann–Whitney U-test (α = 0.05). All tests were performed using the Statistical Package for the Social Sciences^®^ Statistics for Windows version 28 (IBM, Armonk, New York, United States).

## Results

### Fungicidal efficacy of moist heat treatment

**Figure-1:**
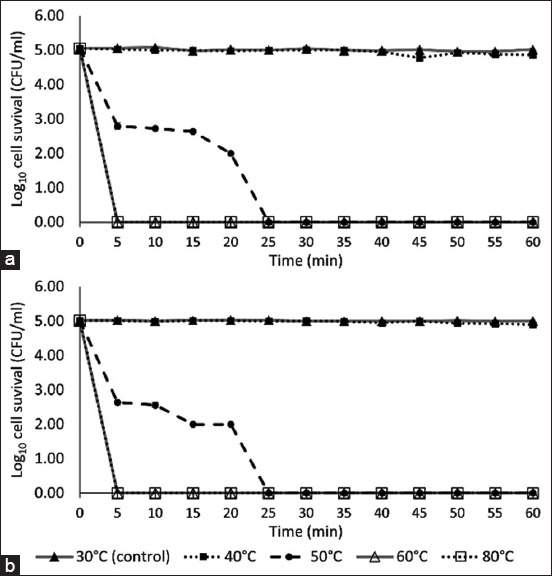
The effect of moist heat treatment against *Microsporum gallinae* American type culture collection 90749 (a) arthroconidia (b) and mycelia. Values represent the means of triplicate experiments with error bars (standard deviation).

The effect of moist heat treatment on *M. gallinae* arthroconidia and mycelia is shown in Figure-1. Arthroconidia and mycelia showed similar susceptibility to moist heat treatment (p > 0.05). Moist heat treatment at 30°C and 40°C did not reduce the number of viable fungal cells after 60 min of contact. However, treatment at 50°C reduced the number of viable fungal cells by 99% (2-log_10_ reduction) at 5–15 min, 99.9% (3-log_10_ reduction) at 20 min, and 99.999% (5-log_10_ reduction) at ≥ 25 min. No viable fungal cells were recovered from 5 to 60 min (99.999% or 5-log_10_ reduction) in tubes treated at 60°C and 80°C.

### Fungicidal efficacy of UV irradiation

**Figure-2:**
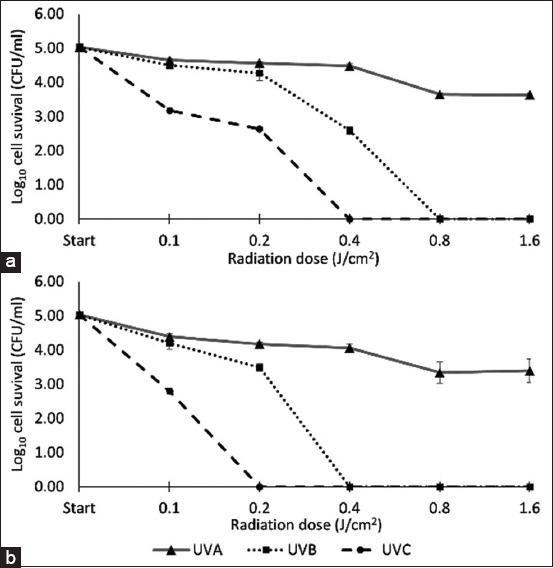
The effect of ultraviolet irradiation against *Microsporum gallinae* American type culture collection 90749 (a) arthroconidia (b) and mycelia. Values represent the means of triplicate experiments with error bars (standard deviation).

While UV irradiation at 365 nm (UVA), 302 nm (UVB), and 254 nm (UVC) showed varying fungicidal efficacy against *M. gallinae* ATCC 90749, there was no statistical difference between its efficacy in killing arthroconidia and mycelia (p > 0.05). Figure-2 shows that the highest energy UVC irradiation was the most effective, with radiation doses of 0.4 and 0.2 J/cm^2^ reducing the number of viable arthroconidia and mycelia by 5-log_10_ respectively. Medium energy UVB irradiation showed lower antifungal activity, requiring 0.8 and 0.4 J/cm^2^ of UVB doses to reduce the number of viable arthroconidia and mycelia by 5-log_10_. The lowest energy UVA irradiation showed the least antifungal activity and a dose of 1.6 J/cm^2^ was unable to reduce the number of viable fungal cells by > 2-log_10_.

### Fungicidal efficacy of commercially available detergents

**Figure-3:**
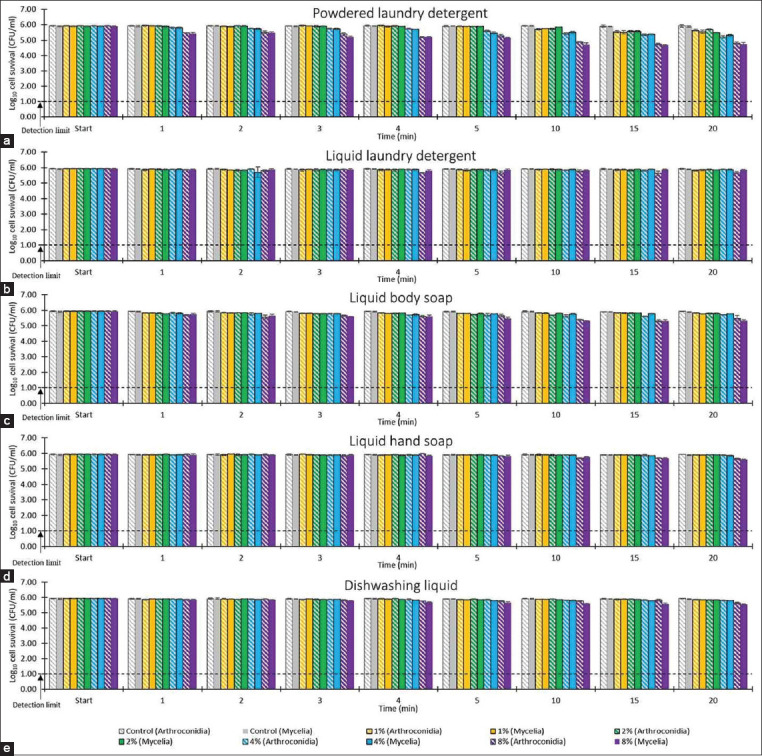
The effect of commercially available detergents (1-8% w/v) against *Microsporum gallinae* American type culture collection 90749 arthroconidia and mycelia; (a) powdered laundry detergent, (b) liquid laundry detergent, (c) liquid body soap, (d) liquid hand soap and (e), dishwashing liquid. Control=phosphate-buffered saline pH 7.2. Values represent the means of triplicate experiments with error bars (standard deviation).

None of the tested commercially available detergents showed effective fungicidal activity against *M. gallinae* ATCC 90749 (Figure-3). Arthroconidia and mycelia showed similar resistance to the killing action of each agent (p > 0.05). Only 8% w/v powdered laundry detergent showed any antifungal effects, slightly reducing the number of viable fungal cells by 1-log_10_ at 10–20 min. In contrast, all other agents reduced the number of viable fungal cells by < 1-log_10_ at 20 min.

### Antifungal efficacy of commonly used germicides

#### Broth microdilution

Table-3 shows the MIC and MFC results of the broth microdilution testing of the selected germicides against the arthroconidia and mycelia of *M. gallinae* ATCC 90749. The selected germicides showed equivalent MICs and MFCs for both arthroconidia and mycelia. However, hydrogen peroxide and phenol were more active against mycelia than against arthroconidia, and the MFCs for sodium hypochlorite were one dilution higher than their corresponding MICs. Chlorhexidine showed the highest antifungal activity (MIC and MFC of 0.195 µg/mL), followed by benzalkonium chloride (1.563 µg/mL), formaldehyde (6.250 µg/mL), glutaraldehyde (25.000 µg/mL), and povidone-iodine (400 µg/mL). Sodium hypochlorite showed the same MIC (1,600 µg/mL) and MFC (3,200 µg/mL) values against arthroconidia and mycelia. Hydrogen peroxide and phenol showed similar activity against arthroconidia (MIC and MFC of 1,600 µg/mL); however, their respective MICs and MFCs against mycelia were 4- to 8-fold lower. Ethanol showed the lowest antifungal activity, with MIC and MFC of 400 µL/mL, which corresponds to 315,600 µg/mL.

**Table 3:** MICs and MFCs of the germicides against *Microsporum gallinae* ATCC 90749.

Germicide	Antifungal activity			

	Arthroconidia		Mycelia	
	
	MIC	MFC	MIC	MFC
Benzalkonium chloride (mg/mL)	1.563	1.563	1.563	1.563
Chlorhexidine (mg/mL)	0.195	0.195	0.195	0.195
Ethyl alcohol (mL/mL)	400.000	400.000	400.000	400.000
Formaldehyde (mg/mL)	6.250	6.250	6.250	6.250
Glutaraldehyde (mg/mL)	25.000	25.000	25.000	25.000
Hydrogen peroxide (mg/mL)	1,600.000	1,600.000	200.000	400.000
Phenols (mg/mL)	1,600.000	1,600.000	400.000	400.000
Povidone-iodine (mg/mL)	400.000	400.000	400.000	400.000
Sodium hypochlorite (mg/mL)	1,600.000	3,200.000	1,600.000	3,200.000

Values represent the MIC and MFC collected from triplicate experiments. MIC=Minimum inhibitory concentration, MFC=minimum fungicidal concentration, ATCC=American type culture collection

#### Time-kill assay

**Figure-4:**
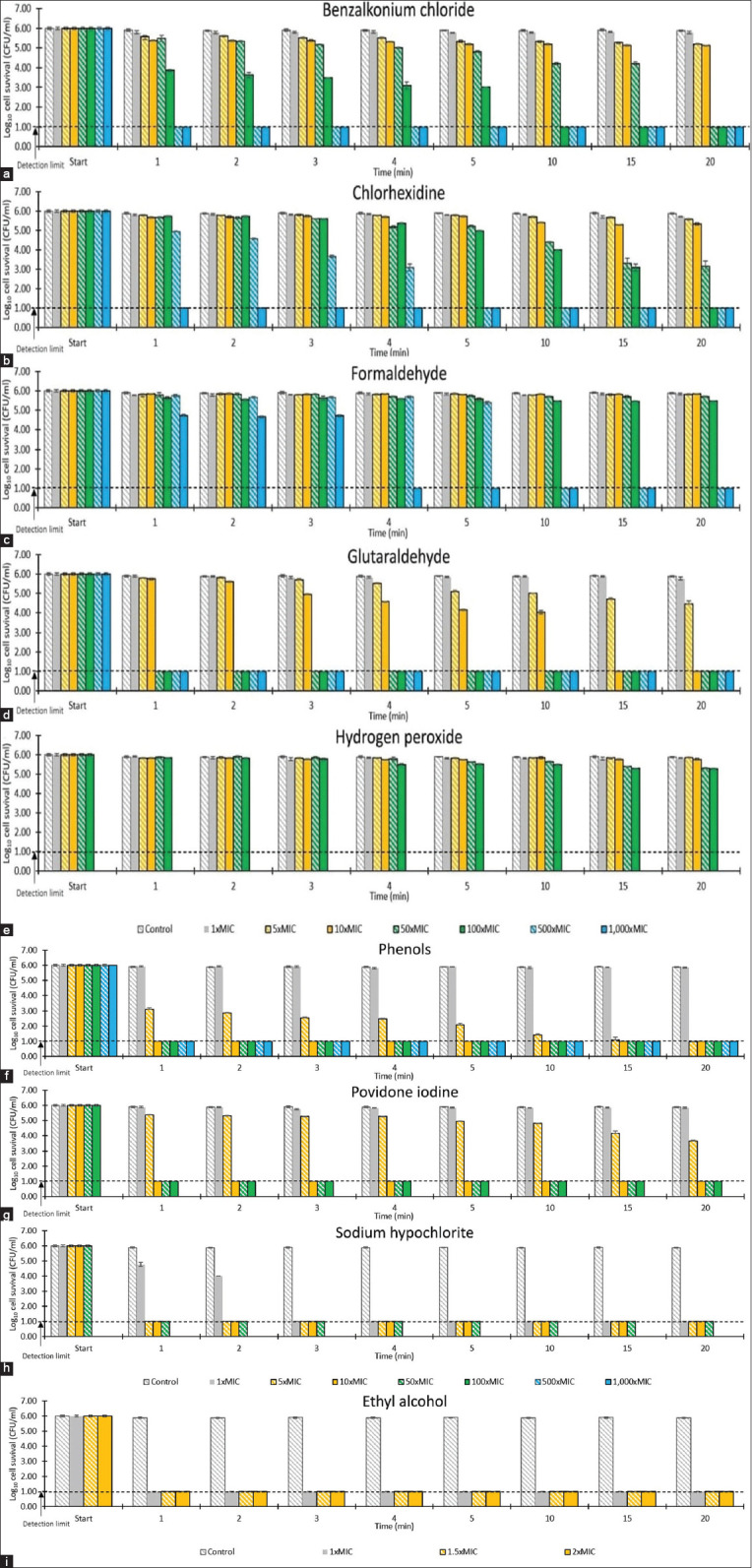
The effect of commonly used germicides against *Microsporum gallinae* American type culture collection 90749 arthroconidia; (a) benzalkonium chloride (1× to 1,000× MIC; 1× MIC=1.563 µg/mL), (b) chlorhexidine (1× to 1,000× MIC; 1× MIC=0.195 µg/mL), (c), formaldehyde (1× to 1,000× MIC; 1× MIC 6.250 µg/mL), (d), glutaraldehyde (1× to 1,000× MIC; 1× MIC=25.000 µg/mL), (e), hydrogen peroxide (1× to 100× MIC; 1× MIC=1,600.000 µg/mL), (f), phenols (1× to 500× MIC; 1× MIC=1,600.000 µg/mL), (g), povidone-iodine (1× to 100× MIC; 1× MIC=400.000 µg/mL), (h), sodium hypochlorite (1× to 50× MIC; 1× MIC=1,600.000 µg/mL), (i), and ethyl alcohol (1×, 1.5×, and 2× MIC 1× MIC=400.000 µL/mL). Control=Neutralizing solution (0.6% w/v sodium thiosulfate, 0.5% w/v polysorbate 80, and 0.07% w/v lecithin in phosphate-buffered saline pH 7.2). Values represent the means of triplicate experiments with error bars (standard deviation). MIC=Minimum inhibitory concentration.

The time-kill assay was conducted to evaluate the selected germicides’ efficacies against *M. gallinae* ATCC 90749 arthroconidia at concentrations ranging from 1- to 1000-fold of their respective MICs (exposure time: Up to 20 min) (Figure-4). Benzalkonium chloride required a concentration of at least 50-fold its MIC to eradicate the fungus. At 50-fold the MIC (78.15 µg/mL), benzalkonium chloride showed a < 2-log_10_ reduction in the number of viable arthroconidia at 15 min but reduced the number of fungal cells by > 5-log_10_ at 20 min. Higher concentrations of benzalkonium chloride were more effective, with no viable arthroconidia recovered after exposure to 100-fold the MIC concentration (156.3 µg/mL) for 10 min and exposure to 500- and 1000-fold the MIC (781.5 µg/mL and 1563 µg/mL) for 1 min (> 5-log_10_ reduction). Arthroconidia were eliminated by exposure to 100-fold the MIC of chlorhexidine (19.5 µg/mL) for 20 min, 500-fold the MIC (97.5 µg/mL) for 5 min, and 1000-fold the MIC (195 µg/mL) for 1 min. With regard to formaldehyde, arthroconidia were eliminated after 10 min at 500-fold the MIC (3,125 µg/mL) and 4 min with 1000-fold the MIC (6,250 µg/mL). Glutaraldehyde at 10-fold the MIC (250 µg/mL) reduced the number of viable arthroconidia by > 5-log_10_ at 15 min and eliminated the fungus after 1 min at 50- to 1000-fold the MIC (1,250–25,000 µg/mL). Exposure to ≥ 10-fold the MIC of phenol (16,000 µg/mL) and povidone-iodine (4,000 µg/mL) reduced the number of viable arthroconidia by >5-log_10_ after 1 min. Sodium hypochlorite at the MIC (1,600 µg/mL) reduced the number of viable fungal cells by > 5-log_10_ at 3 min and within 1 min at concentrations of ≥ 5-fold the MIC (8,000 µg/mL). Ethanol showed a marked antifungal effect, decreasing the number of viable fungal arthroconidia by > 5-log_10_ within 1 min at the MIC (400 µL/mL). Conversely, the highest tested concentration of hydrogen peroxide (100-fold the MIC, 160,000 µg/mL) did not reduce the number of viable arthroconidia by > 1 log_10_ at 20 min.

## Discussion

The testing of disinfection processes in this study demonstrates new data that have not been revealed previously, particularly the time-kill kinetics of the disinfection process specific to *M. gallinae* arthroconidia and mycelia. The results revealed that moist heat treatment, UV irradiation, and germicides were effective methods suitable for application in poultry husbandry. However, the routine use of commercially available detergents is unlikely to affect fungal viability. The reproductive (arthroconidia) and vegetative (mycelia) growth stages of *M. gallinae* showed similar sensitivity to moist heat, UV irradiation, and germicide treatments, indicating that the same processes can be used to decontaminate both arthroconidia and mycelia from equipment. The fungus *M. gallinae* is a member of the zoophilic dermatophytes, and asexual spores or arthroconidia develop from hyphae when conditions are unsuitable for growth and survival. The immune response mounted by the host in *M. gallinae* lesions reduces O_2_ and increases CO_2_, stimulating fungal hyphae to develop into arthroconidia [12]. Arthroconidia are *M. gallinae* spores can spread from infected animals to the environment and other hosts. Spores represent the most important infective stage of dermatophyte species due to their extended viability and increased virulence. Similarities or differences in the sensitivity of spores and mycelia to disinfection processes depend on the self-defense structures and processes present in individual fungal species and strains [10, 17].

In this study, effective decontamination with moist heat was achieved at temperatures of > 50°C, with *M. gallinae* ATCC 90749 being particularly sensitive to temperatures of ≥ 60°C, requiring only 5 min to reduce the number of viable cells by 99.999% (5-log_10_). In contrast, conidiospores (macroconidia and microconidia) of the related dermatophytes *Trichophyton mentagrophytes*, *Trichophyton rubrum*, and *Epidermophyton floccosum* were more resistant to moist heat, requiring 16 min and 20 min for 5-log_10_ reduction in the number of viable *E. floccosum* and *T. mentagrophytes* and *T. rubrum*, respectively, at 80°C [18]. This difference in moist heat tolerance is due to the differential expression of heat shock proteins (HSPs), which facilitate microorganism resistance to temperature shifts, toxic chemicals, and other harsh environmental conditions. HSPs are important protective agents that are rapidly activated after exposure to adverse environmental conditions to protect cells from denatured protein aggregates; they are involved in pathogenicity, virulence, organism’s life cycle, survival under stress, and resistance to antifungals [19].

Conventional cooking processes generate heat higher than 80°C, which is sufficient to decontaminate *M. gallinae* on heat-resistant equipment and tools. In addition, applying a wash temperature of > 60°C for laundry is likely to be suitable to control the spread of *M. gallinae* on farmworker’s clothes. For poultry manure management, heat treatment has advantages over disinfectants because poultry manure is rich in biomaterials and germicides are less effective under such conditions. Quicklime treatment is one of the most common disinfection methods for animal manure and sewage sludge; this process mixes quicklime (calcium oxide) with manure at a rate of 10–20% by weight and the resulting hydration and exothermic reactions increase the pH to 11–12 and the temperature to 55–70°C [20, 21].

UV radiation has been used for disinfection since the late 19^th^ century as it has a wide spectrum of antimicrobial activity. It can be used to eradicate bacteria, fungi, viruses, and bacterial spores. The germicidal activity of UV radiation depends on the wavelength used (shorter wavelengths have higher energy and higher activity) and radiation dose (intensity and duration) applied [22]. UV radiation kills microorganisms by causing DNA and RNA damage and initiating photosensitization and oxidation reactions in cells [23, 24]. This study shows that irradiation with shorter wavelength UVC (100–280 nm) and UVB (280–315 nm) at doses of 0.4 and 0.8 J/cm^2^, respectively, was effective at eradicating *M. gallinae*. In contrast, UVA (315–400 nm) was unable to reduce the number of fungal cells by > 99% at the highest dose. A previous study conducted by Dai *et al*. [25] indicated that different dermatophyte species and strains have different sensitivities to UV radiation. In one study, *Microsporum canis*, *T. rubrum*, *T. mentagrophytes*, and *E. floccosum* exposed to 0.12 J/cm^2^ UVC showed reductions of 99.99%, 99.9%, 99.9%, and 99%, respectively, in the number of viable fungal cells. Another study conducted by Nematollahi *et al*. [14] found that UVB and UVC showed similar activities against *T. mentagrophytes* and *T. rubrum* at 0.12 J/cm^2^, reducing the number of viable cells by 39-76% and 59–80%, respectively, whereas UVA at 10-fold the dose (15 J/cm^2^) showed only a 15%-73% and 84%–88% reduction in *T. mentagrophytes* and *T. rubrum*, respectively.

The detergents tested in this study contain various surfactants as their main constituents and did not contain disinfectants, except chloroxylenol in liquid hand soap. These products represent commercial detergents for routine home and farm applications. Some surfactants have been reported to act against pathogens such as *Staphylococcus aureus*, *Escherichia coli*, *Bacillus subtilis*, and *Candida albicans* by damaging the cytoplasmic membrane to cause leakage of intracellular material and by inducing protein and nucleic acid degradation [26]. However, the present study reveals that detergents showed only slight fungicidal activity against *M. gallinae* at a concentration of 8% w/v, which is substantially higher than the routine practical concentration; thus, these products cannot be used for fungal disinfection. Nevertheless, in normal laundry practice, these detergents will wash away large fungal materials and other biomaterials from clothes and reduce the amount of other contaminating microorganisms, which would promote the efficacy of subsequent disinfection processes [27].

This study shows that the tested germicides were effective against *M. gallinae* with a wide range of MIC values. The tested germicides are widely used both in medical and farm practices. Benzalkonium chloride, chlorhexidine, ethanol, hydrogen peroxide, and povidone-iodine are antiseptic and disinfectant agents that can be used to disinfect both living tissue and non-living objects. Formaldehyde, glutaraldehyde, phenol, and sodium hypochlorite are unsafe for use with living tissue and can be used only on inanimate objects and environments. Some previous studies have investigated the efficacy of these germicides against other dermatophyte species. Gomes *et al*. [28] reported that chlorhexidine digluconate had a MIC of 4.41 mg/mL (equivalent to 2.48 mg/mL chlorhexidine) against *M. canis* and *Microsporum gypseum*. In comparison, sodium hypochlorite had MICs of 11.1–44.4 mg/mL and 11.11–88.88 mg/mL against *M. canis* and *M. gypseum*, respectively. Perrins *et al*. [29] found that the chlorhexidine MIC range against *T. mentagrophytes* and *Microsporum persicolor* was 18.75–50 µg/mL, and against *Trichophyton erinacei* it was 12.5–50 µg/mL. Eloff *et al*. [30] showed that the MIC of ethanol against *M. canis* was 163 µL/mL and was in the range of 328–411 µL/mL against pathogenic fungi such as *C. albicans*, *Cryptococcus neoformans*, and *Sporothrix schenckii*.

According to the guidelines of the Association of Official Analytical Chemists [11], the appropriate germicide concentration that should be applied to achieve effective disinfection of inanimate surfaces is the lowest concentration capable of killing 10^5^ conidia/mL within 10 min. Therefore, the time-kill kinetic results of the present study indicate that the optimal concentrations of germicides that should be applied for effective disinfection of *M. gallinae* from surfaces are as follows: benzalkonium chloride, 156.3 µg/mL; chlorhexidine, 97.5 µg/mL; ethanol, 400 µL/mL; formaldehyde, 3,125 µg/mL; glutaraldehyde, 1,250 µg/mL; phenol, 16,000 µg/mL; povidone-iodine, 4,000 µg/mL; and sodium hypochlorite, 1,600 µg/mL. Hydrogen peroxide showed limited activity against *M. gallinae*, and there is no recommended concentration for effective disinfection of *M. gallinae* from surfaces using hydrogen peroxide. However, in field practice conditions, the presence of contaminating organic matter in the environment can profoundly affect the efficacy of some disinfectants, which may necessitate the use of higher concentrations, especially for disinfectants based on the activity of chlorine and iodophors, and other oxidizing disinfectants. In addition, water hardness can directly affect the effectiveness of iodophor- and benzalkonium chloride-based disinfectants [10].

## Conclusion

The susceptibility of arthroconidia and mycelia of *M. gallinae* to all tested disinfection processes was similar. We recommend moist heat treatment at 50°C for 25 min or ≥ 60°C for 5 min and irradiation with UVC at 0.4 J/cm^2^ and UVB at ≥ 0.8 J/cm^2^ for the control of *M. gallinae* in poultry husbandry, based on the results of our study. The chemical disinfectants benzalkonium chloride (156.3 µg/mL), chlorhexidine (97.5 µg/mL), ethanol (400 µL/mL), formaldehyde (3,125 µg/mL), glutaraldehyde (1,250 µg/mL), phenol (16,000 µg/mL), povidone-iodine (4,000 µg/mL), and sodium hypochlorite (1,600 µg/mL) showed an eradicating effect against *M. gallinae* arthroconidia, decreasing the number of viable cells by > 99.999% within 10 min. Hydrogen peroxide, powdered laundry detergent, liquid laundry detergent, liquid body soap, liquid hand soap, and dishwashing liquid are unsuitable for use as *M. gallinae* disinfectants but may be used for cleaning purposes.

## Authors’ Contributions

ET: Performed the experiments of antifungal testing of disinfection processes, performed the statistical analysis, and wrote the manuscript. SJ and SU: Designed the study and contributed to the conception. GNB: Contributed to the manuscript draft and conducted grammar review. JA: Prepared the fungal samples and participated in experimental design. All authors have read and approved the final manuscript.
